# Sensory Stimuli to Sustainable Social Wellbeing: A Multimodal Approach Based on Warm Scent

**DOI:** 10.3390/bs12050146

**Published:** 2022-05-16

**Authors:** Sinae Lee, Dongwon Min

**Affiliations:** College of Business, Dankook University, 152, Jukjeon-ro, Yongin 16890, Gyonggi, Korea; snlee@dankook.ac.kr

**Keywords:** prosocial behavior, warmth, need for social connectedness, actual scent, simulated scent, multimodality

## Abstract

Sensory experiences affect individuals’ judgment and behavior through the metaphors that experiences carry. The literature has demonstrated that the perception of warmth activates concepts related to positive meaning and metaphors, such as consideration and gentleness, which increase individuals’ tendency to help or relate to others. This study hypothesized that warm olfactory stimuli influence intention toward prosocial behavior by increasing the need for social connectedness (NSC). The first experiment (*n* = 123) demonstrated that the actual warm scent increased participants’ intentions for prosocial behavior and that the effect of the actual warm scent was mediated by NSC. Using Amazon Mechanical Turk (MTurk), a second experiment (*n* = 995) was conducted the showed that warm scent simulated via visual stimuli (i.e., a multimodal approach) led to prosocial behavior intention as well. The results of the study provide academic and managerial insights into how to improve prosocial behavior intention, which is essential for the sustainable development of societies.

## 1. Introduction

In order to build a sustainable future, many companies have been developing businesses with the aim of prosocial management. Companies sympathize with environmental issues such as fine dust, plastic waste, and global warming. They practice prosocial management through eco-friendly marketing in order to curtail these problems, which positively influences society by stimulating responsible consumption and by establishing new management strategies that contribute to brand reputation. This trend is now lead by global brands such as Volvo, Prada and Burberry [1], and without doubt, prosocial management is now considered a topic that must be discussed constantly in order to promote long-term business activity, organizational sustainability, and social development.

As such, companies have been focusing on becoming the main agent of solving various problems that arise in society along with the development of companies through pro-social activities that pursue their own interests and social values at the same time. However, in many cases, the active participation of a consumer, which contains inconveniences such as ‘using paper/starch straws instead of plastic straws’, is needed to perform prosocial management. Even if people recognize that participating in a campaign is an action that results in a more social and reciprocal community, if participating in it requires additional costs like spending an particular amount of their time, putting in extra effort, or behavioral changes, consumers will not likely be wanting to participate in the campaign [2]. For this reason, many companies, as well as various environmental and civic groups, have led promotions in various ways to shift consumer awareness and induce prosocial behavior. Nevertheless, inducing action beyond the perception of “doing the right thing” is difficult.

Meanwhile, numerous studies have recently demonstrated that consumer behavior can be possibly induced by using relatively less visible and nonconscious methods such as sensory stimulation. This is because sensory stimulation activates concepts associated with metaphors, which is a method used to linguistically describe intangible and abstract concepts referring to another similar object [3], thereby influencing when judging or choosing a subject. Examples include using “warm” to convey abstract concepts of intimacy or consideration or “soft” to explain ease or facility. However, as in the previous example of experiments, bodily states sometimes elicit metaphor-related concepts in sensory processing [4]. For example, chocolate eaters may assess themselves and others to be more agreeable [5], and a heavier book may be deemed to be more important than a lighter one [6]. These examples demonstrate that experiences of sensing sweetness and heaviness activate the concepts of affability and importance, which unconsciously influence one’s judgments and choices. This study explores metaphors related to warmth, and in particular, the olfactory stimuli that are rarely used to analyze how the sense of warmth would lead to warmth-oriented judgments and choices. Metaphors of warmth positively affect social connectedness [7] because concepts, such as gentleness, closeness, and consideration embodied in such metaphors increase individuals’ tendency to help or relate to others [8]. Additionally, it also argued that an increased need for social connectedness (NSC) has a positive effect on prosocial behavior, such as donating to charities or participating in voluntary services [9]. Therefore, we hypothesized that individuals who experienced a scent associated with warmth would have a higher willingness to participate in prosocial activities or purchase cause-related products than those who did not.

One pretest and two main experiments were conducted based on this hypothesis, with the following procedures: we observed whether olfactory experience associated with warmth-related metaphors led to prosocial behavior. We also explored whether this process is mediated by a need for social connection. Finally, we examined whether the results were the same for a warm scent simulated via visual stimuli in the absence of an actual scent (i.e., a multimodal approach).

## 2. Literature Review

### 2.1. Social Warmth and Sustainable Wellbeing

Social wellbeing is of paramount importance at present, especially in the face of the global crisis caused by COVID-19 [10,11]. It is important as this crisis influences individuals both physically and mentally [11] and is closely related to maintaining a positive attitude toward others [12,13,14]. Now, what are the factors necessary for sustainable social wellbeing in the current global context? There are various factors, such as social interaction and social capital [15]; however, the improvement of social relationships is the most important of them all [16,17].

One of the ways of improving social relationships is by enhancing social warmth. This is due to the role of social warmth in inducing action for the welfare of people, including altruistic actions (e.g., prosocial actions and reciprocal actions). It is important to note that social warmth can be triggered through physical warmth. Physical warmth can be felt through direct contact with the temperature of the surrounding air or a specific object, and this perceived warmth leads to motivation to form a bond with others. For example, subjects seated in a warm chair felt a greater need for social belonging and bonding than subjects who did not [18], and the higher the temperature of the object the subjects held in their hand, the more they perceived themselves as having a need for social connection [19,20]. Physical warmth also promotes generosity or closeness to others. After drinking a hot drink, you can evaluate your partner more generously [21], and a store’s warm, rather than cold, lighting can generate a higher perception of closeness toward the store [22]. This is because the sensory experience of warmth triggers the activation of a concept related to the metaphor of warmth (e.g., closeness, kindness, caring, etc.), leading people to an action associated with the concept. These are all findings from previous research that this study is based on. In particular, we will proceed with this study from a metaphorical perspective, focusing on the sense of smell as it operates in collaboration with other senses. As we will see, warm stimuli raise the need for social connectedness (NSC) [19,20,23], which is the main factor for the increase in expressing and perceiving social warmth.

### 2.2. Metaphor and Olfactory Stimuli

People often use metaphorical expressions to describe ambiguous notions, such as social relationships and human characteristics. Hence, “warm” people always feel “close” to others and their conversations are mostly described as “sweet.” Conversations often incorporate expressions using temperature, spatial distance, or taste to facilitate understanding. This is a method of connecting the target to more concrete sources acquired from sensory experience to facilitate the understanding of abstract concepts [24], such as personalities, morality, social relationships, or suspicion. For example, the sense of smell is metaphorically associated with the concept of suspicion [25,26]. Smell, which is an evolutionary mechanism, has long been linked to the understanding that rotten food may endanger life; therefore, a substance that smells bad is suspicious [24]. Thus, individuals have learned from experience that smell is linked to suspicion, which is demonstrated by using the idiomatic expression “something smells fishy.”

Likewise, in situations related to a disconnection of interpersonal interaction, such as loneliness or exclusion, metaphorical expressions related to the sense of “cold” are often used. This is because individuals learn from an early age that being socially distant from others is associated with the physical experience of being relatively less warm. Studies on judgment and choice observing the metaphorical concepts activated by sensory experiences show that physical coldness increases loneliness (i.e., social coldness) [27]. The mere act of touching sandpaper’s coarse texture may make people perceive their relationships with others to be rougher (i.e., difficult and hostile) [28]. Some studies anticipate that sweet-tasting experiences will make others feel more agreeable [5] and that those who enjoy a spicy taste will be more temperamental than those who enjoy sweet tastes [29].

Over the last decade, studies on concepts related to metaphors activated by olfactory cues have increased. The feeling of suspicion has been found to be activated in groups who had experienced fishy odors, and therefore, such groups processed information in a more critical and meticulous manner [24]. In another study, the group that experienced the warm scent of vanilla perceived a space to be populated by more people than groups that had experienced the cold scent of peppermint, which led to an increase in individuals’ capability and intention to purchase luxury goods [30]. In addition, a study by Liljenquist et al. [1] found that scents associated with cleanliness encourage prosocial behavior by activating morality. Likewise, citrus scents increased sensitivity to words related to cleaning, and those breathing the scent strived to keep their surroundings more hygienic [31].

From the above examples, we can infer that sensory experiences that activate metaphor-related concepts may contribute to unconscious behavioral changes in consumers in various directions. However, studies on metaphors related to the sense of smell have been relatively rare compared to those associated with other senses [32], and many studies using olfactory stimuli have focused on memory improvement or satisfaction caused by the presence of the scent itself, or the type, strength, and suitability of the scent. Therefore, in this study, it is noted that among the five senses, the sense of smell has relatively subliminal effects on consumers. The positive effects of activating the concepts related to the metaphor of warmth (e.g., consideration, helpfulness, and kindness) inherent in certain olfactory experiences (e.g., warm scents) are being examined in this paper.

### 2.3. Effects of Warm Sensory Stimulation

Expressions of perceived temperature, such as warmth and coldness, are frequently utilized to describe social relationships or individuals’ abstract features. Words like “to feel as if walking on thin ice” or “cold war” are used to describe negative signs or breakups in interpersonal relationships. In contrast, expressions such as “heartwarming” or “warm helping hand” are used to describe positive signs or affection. As shown before, the perception of physical coldness is highly correlated with feelings of loneliness or social exclusion [27,33]. While the perception of physical warmth can positively influence interpersonal relationships and prosocial behavior, it can also reduce social distance, and foster social connections [21,34,35]. Warm ambient lighting (vs. cold lighting) was also found to drive stronger intentions to make donations [36].

There is a fundamental reason why physical warmth is associated with social warmth. The experience of the former indicates that there is an object that is the source of warmth in physical proximity. For instance, newborn babies experience their mothers’ body warmth during the first moments of their life. In the course of life, close physical contact with caregivers is frequent, accompanied by an experience of the caregiver’s warm body [37]. Spatial constraint is an important factor in perceiving another person’s body temperature, as bodies must be in proximity to transfer warmth [18]. One may perceive an increase in temperature in proximity to another person, even though the actual temperature has not changed at all [23]. Fay and Maner [18] additionally noted that the concepts metaphorically associated with warmth would not be activated if the initial sensory experience of warmth had been insufficient in the early stages of life and that these concepts are acquired through such continued sensory experiences.

These findings demonstrate that neural activity occurs in the same area of the brain when actual physical warmth is applied (e.g., holding heat packs) and when social warmth is experienced (e.g., reading delightful messages from close friends). Hence, social and physical warmth both share the same biological mechanisms [19].

The olfactory sense may also affect metaphors related to temperatures. Warm scents may cause a bias in spatial perceptions (e.g., warmth causes a perception of proximity and therefore, crowding) [30], and cool-scented therapeutic gel-packs may be perceived to be more effective than warm-scented ones.

Therefore, we anticipated that olfactory cues containing temperature-related metaphors can affect the judgment or evaluation of social relationships, as warm scents are sufficient to activate metaphorical concepts related to warmth.

### 2.4. Multimodal Approach to Stimulation

Metaphors arise based on the correlation between sensorimotor and conceptual activation as experiences are accumulated [31,38]. That is, an understanding of metaphors based on accumulated experiences is essential when comprehending the concept related to metaphors. Following from Hong and Sun’s [39] study, it was shown that the consumption of hot tea leads to a preference for romance movies. Specifically, these results were significant when there was an understanding of the metaphor between warmth and romance.

In another experiment, the simulation effect of a sensorimotor experience is significant only when there is an understanding of the concepts related to metaphors. Specifically, participants in Slepian [40] were asked to classify eight grayscale faces as Republicans or Democrats. Half of the grayscale faces were presented next to hard objects (e.g., wooden blocks) and the remaining faces were presented next to soft objects (i.e., cotton balls). The participants then completed a metaphor-comprehension measure (i.e., they were asked to rate the Republicans and Democrats as either as a “hard politician” or as a “soft politician”). The faces next to the soft object were considered softer. However, these results do not appear without an understanding of the metaphor between softness and the Democrats. This makes it clear that an understanding of metaphor is essential when activating metaphor-related concepts, and it can be seen that such an activation is possible without using actual sensory stimulation.

Therefore, in this study, we chose to adopt a multimodal approach that can activate the simulation of olfactory stimulation through visual stimulation by utilizing the proven olfactory stimulation as a stimulus that can lead to a better understanding of the metaphor of warmth.

### 2.5. The Need for Social Connectedness and Prosocial Behavior

When NSC increases due to a sense of belonging emotionally to another person or group [41], individuals attempt to adapt to the organization, respect social norms and cooperate more for social harmony [42]. Previous studies have analyzed the positive effects of social connectedness and prosocial behavior. These studies found that a higher level of social connectedness means a greater inclination to participate in charity or voluntary services [9], and the greater the NSC, the more generous one’s actions are toward others in need [43]. Moreover, the sense of social connection induces more socially responsible behavior [44].

This sense of social connection can be activated through external stimuli, including cues of psychological or physical warmth. Experiencing warmth activates its associated concepts, increasing the tendency to pay more attention to others and to build relationships [7]. Thus, the experience of warmth heightens the need for social connection, in turn inducing prosocial behavior. As has been seen before, the experience of warmth may also be activated by metaphors that evoke a sense of warmth. Therefore, the following hypotheses were formulated:

**H1.**  *The experience of a warm scent will have a positive effect on prosocial behavior.*

**H2.**  *The experience of a warm scent will increase the perception of the NSC.*

**H3.**  *The experience of a warm scent will have a positive effect on prosocial behavior through an increased perception of the need for social connection.*

**Figure 1:**
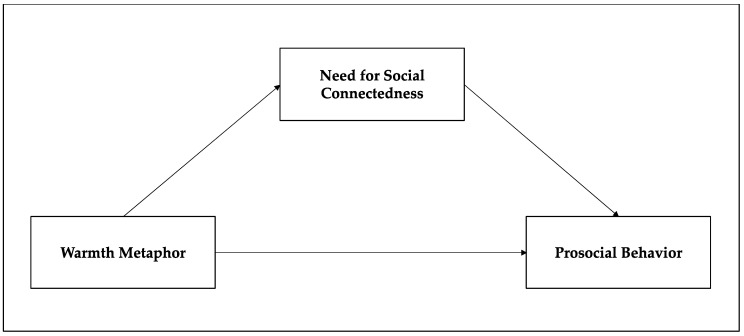
Conceptual Research Model.

**H4.**  *The simulation of olfactory stimulation through visual stimulation will have the same effect as the experience of an actual warm scent (see Figure 1).*

## 3. Materials and Methods

Informed consent was obtained from all human subjects involved in this experimentation and their privacy rights were observed throughout this research study.

The initial step of the experiment was to perform a pretest, the purpose of which was to investigate the positive effects of warm scent experiences on prosocial behavior. Thereafter, in Experiment 1, we examined whether the pretest results were driven by the experience of warm scent activating concepts related to metaphors, using the mediator NSC. In Experiment 2, we investigated whether the mechanisms demonstrated in the pretest and Experiment 1 could be replicated by a multimodal approach that simulated the sense of smell based on sight in the absence of an actual scent.

We also used the peppermint scent in the experiment, as it has been observed to be a popular scent in previous studies [45]. The existing literature has shown that fragrance itself can have a positive effect on consumer behavior [46]. The control group was exposed to no scent in order to derive more reliable results.

### 3.1. Pretest

Many preceding studies have verified the sensory experience of warmth through haptic experiments, and some have examined the effects of concepts activated by warm scents, but examinations into the effects on prosocial behavior have been absent. The pretest explored whether warm scents positively affect prosocial behavior like other sensory experiences (e.g., haptic).

#### 3.1.1. Method

##### Participants and Design

A total of 70 undergraduate students from Dankook University (in Gyunggi-do, Republic of Korea) participated in the pretest (male 36; female: 34; ages 20 to 21 years; Mean = 20.84, Standard Deviation = 1.80). The pretest adopted a between-subjects design (scents: warm vs. cool), with the dependent variable being the intention to participate in prosocial behavior.

##### Procedure and Materials

Two scents were prepared to manipulate perceptions of warmth and coolness. Both warm vanilla and cool peppermint scents were prepared by a perfume company, the same as those used by Madzharov et al. [30] A lecture hall at the DanKook University was prepared by spreading warm and cool scents an hour prior to the subjects’ entry. A cotton cloth was soaked in each scent and was applied on the subjects’ desks, and the two rooms used in the pretest were located on different floors to separate subjects and to thereby prevent the possibility of different scents mixing or influencing the subjects. In addition, the two rooms were maintained at 80.6 °F to exclude the effect of temperature.

**Figure A1:**
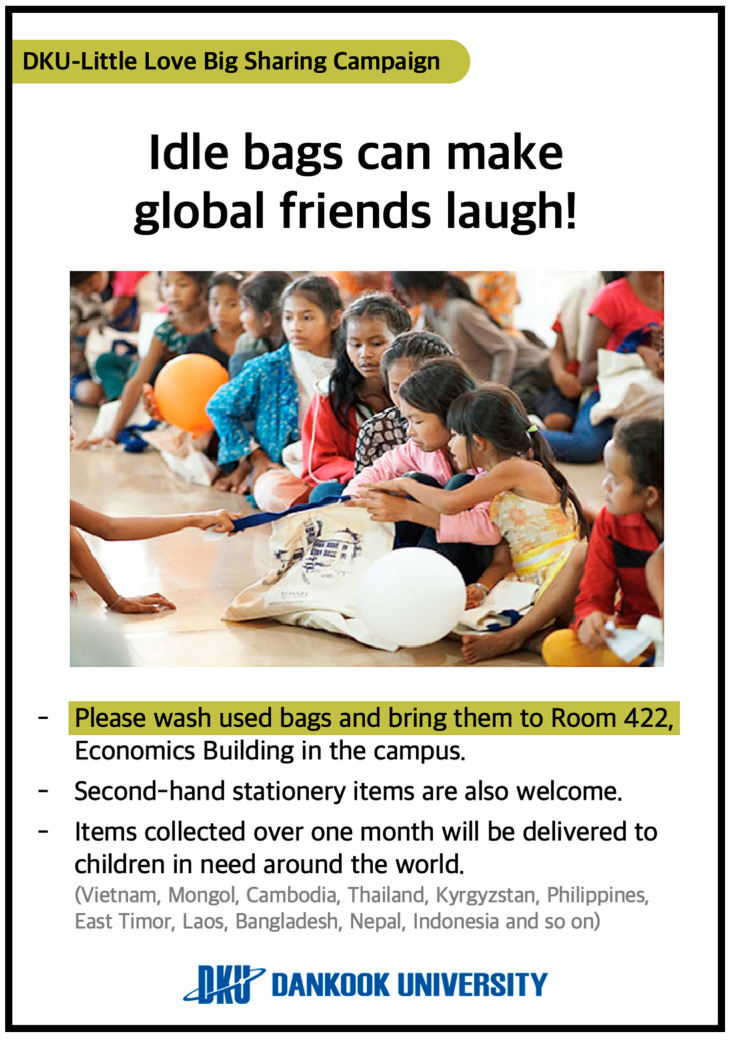
Intention to participate in a bag-donation campaign (Experiment 1).

The prosocial behavior designated as the experimental target was a bag-donation campaign (see Figure A1). The subjects were asked to “wash used bags and bring them to Room 422 of the Economics Building,” with the slogan “DanKook University (DKU) Little Love Big Sharing Campaign.” The following messages were also presented with the university logo: “Idle bags can make global friends laugh”; “Second-hand stationery items are also welcome”; and “Items collected over one month will be delivered to children in need around the world.”

The pretest was conducted for approximately 10 min. After the subjects viewed a short video that was irrelevant to the pretest, they were presented with an advertisement of the DKU Little Love Big Sharing Campaign. The subjects first responded regarding their intention to participate in the campaign using a seven-point scale (−3 = no intention to participate vs. 3 = strong intention to participate). This was followed by a survey of the perceived room temperature on a seven-point scale (−3 = cool vs. 3 = warm). According to the existing literature, we also confirmed the age and gender of the participants as there could be a difference in donation intention depending on age and gender [47]. Finally, in order to confirm the mood effect caused by the scent itself, the moods of the subjects were also measured using a seven-point scale (−3 = not good vs. very good). After all the experimental procedures were completed, the subjects were informed that the actual purpose of the pretest was to “explore the effect of warm scents on intentions toward prosocial behavior.”

#### 3.1.2. Results

##### Manipulation Check

A manipulation check for 70 subjects demonstrated that there were differences in perceived temperature (M_warm_ = 5.43 vs. M_cool_ = 2.14; *t*(68) = 10.96, *p* < 0.001, CI = 2.68, 2.88), indicating a successful manipulation of temperature using different scents (warm vs. cool).

##### Experimental Results

A t-test was conducted with a warm (vs. cool) scent as the independent variable and intentions toward prosocial behavior as the dependent variable. The results demonstrated greater intentions to participate in prosocial behavior in the group exposed to the warm (vs. cool) scent (M_warm_ = 5.51 vs. M_cool_ = 4.83; *t*(68) = 2.47, *p* < 0.01, CI = 0.13, 1.24). The effect size was calculated using “the Cohen’s D,” [48] Cohen’s *d* = 0.59 (<0.05, middle). The effects of age, gender, and mood were confirmed through ANCOVA (Analysis of covariance), and the results were not significant (*p*s > 0.1).

#### 3.1.3. Discussion

The pretest demonstrated that the experience of warmth induces the intention to show prosocial behaviors, as was also presented in previous studies [21]. Furthermore, such a change in behavior may be achieved by the sense of touch and smell (i.e., scent). However, it was unclear whether this effect was caused by the activation of concepts related to metaphors triggered by a sensory experience of warmth. Therefore, in Experiment 1, we particularly examined the process by which warm scents induced prosocial behavior.

### 3.2. Experiment 1

To ensure that the positive effects of warm scents on prosocial behavior were based on concepts (i.e., consideration, altruism, friendliness, etc.) activated by metaphors associated with warmth, the following mediator was added: NSC. This is because, if warm scents trigger intentions toward prosocial behavior by activating concepts associated with relevant metaphors, then, in turn, the NSC should also increase, because concepts related to warmth increase the NSC [7].

#### 3.2.1. Methods

##### Participants and Design

A total of 123 undergraduates of Dankook University (male: 52; female: 71) participated in the experiment (ages 18 to 33 years; M = 19.77, SD = 2.48). The experiment adopted a between-subjects design (scents: warm vs. cool vs. control). The dependent variable was intention toward prosocial behavior, and the mediator was NSC.

##### Procedure and Materials

To manipulate perceptions of warmth and coolness, the vanilla and peppermint scents used in the pretest were reused with the same procedures. The experiment for the control group was conducted in an odorless lecture hall. To exclude the effects of existing room odors, all rooms (odorless, warm- and cool-scented ones) were ventilated for 1 h before the ambient temperature was set again to 80.6 °F. Stimuli for the experiment were identical to those of the pretest, and the experiment likewise spanned 10 min. Prior to the experiment, the subjects viewed a short video that was irrelevant to the experiment and were then presented with an advertisement of the DKU Little Love Big Sharing Campaign. The subjects first responded to their intention to participate in the campaign by using a seven-point scale (−3 = no intention to participate vs. 3 = strong intention to participate), and then they responded to eight questions on social connectedness, designed on the basis of the research conducted by Lee and Robins [41]. Specifically, responses on a seven-point scale (−3 = not at all vs. 3 = very much so) were provided for the following statements: “I have a good relationship with people around me”; “I often feel included by the people around me”; “I feel somewhat distant when I am with people”; “I feel together with my friends”; “I do not feel connected with people around me”; “My friends feel like brothers or sisters”; “I am included among friends or various groups”; and “I have a good interpersonal network” (α = 0.95). This was followed by a survey on the perceived room temperature on a seven-point scale (−3 = cool vs. 3 = warm). In the process, the questionnaires of three unfaithful respondents were removed. Finally, the subjects were asked to provide their age and gender. After all the experiments were completed, we briefed the subjects and thanked them for their participation.

#### 3.2.2. Results

##### Manipulation Check

The results of a one-way analysis of variance (ANOVA) for the manipulation check for the 123 subjects showed differences in perceived temperature according to the experienced scent (M_warm_ = 5.58, SD = 0.93 vs. M_cool_ = 2.35, SD = 1.21 vs. M_control_ = 3.25, SD = 1.22; *F*(2, 120) = 89.81, *p* < 0.001, CI = 3.46, 4.09). According to the planned contrast conducted to determine specific differences between groups, the warm group perceived the room temperature as being higher than the cool group [t(120) = 12.88, *p* < 0.001] and the control group [t(120) = 9.45, *p* < 0.001], and the cool group perceived the room temperature lower than did the control group [t(120) = 3.50, *p* < 0.001]. Thus, it was confirmed that scent influenced the perception of warm or cool temperature.

##### Experimental Results

**Figure 2:**
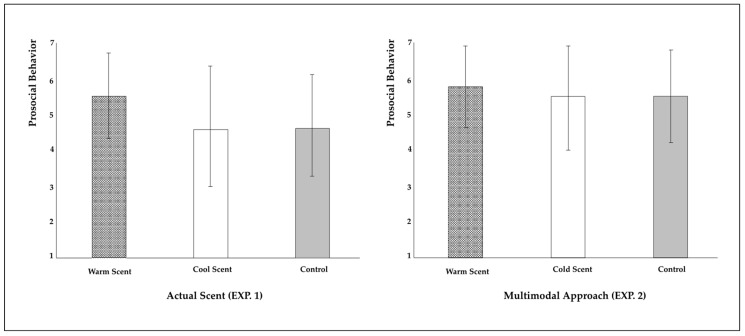
Results of ANOVA (EXP.1 and EXP. 2). Error bars represent one SD.

To determine the results, a one-way ANOVA was conducted with scent (warm vs. cool vs. control) as an independent variable and prosocial behavioral intention as a dependent variable. The results demonstrated greater intention to participate in prosocial behavior in the group exposed to the warm scent (M_warm_ = 5.54, SD = 1.16 vs. M_cool_ = 4.60, SD = 1.65 and M_control_ = 4.62, SD = 1.39; *F*(2, 120) = 4.83, *p* < 0.001, CI = 4.81, 5.29; see Figure 2). According to the planned contrast conducted to determine specific differences between groups, the warm group’s response regarding prosocial behavior intention was higher than that of the cool group [t(120) = 2.68, *p* <.01, Cohen’s *d* = 0.66 (< 0.05, middle)] and the control group [t(120) = 2.66, *p* <.01, Cohen’s *d* = 0.72 (< 0.05, middle)]. The difference between the cool group and the control group was not significant (*p* > 0.1).

Warm scent was designated as an independent variable (with subjects in the warm group coded as 1 and subjects in the cool group as −1), intention to participate in prosocial behavior was the dependent variable, and NSC was the mediator. The number of samples re-extracted for bootstrap was 10,000, and the lower-level confidence interval (LLCI) and upper-level confidence interval (ULCI) of the mediation effect coefficient were obtained from a 95% confidence interval (CI). The indirect effects of NSC demonstrate that zero was not included between the LLCI and ULCI, which were 0.03 and 0.37, respectively, thus verifying statistical significance (For direct effect, see Table 1). Hence, NSC’s mediation effect on warm scents’ influence on intentions toward prosocial behavior was verified (see Table 2). The LLCI and ULCI were also calculated for the cool vs. control groups (with subjects in the cool group coded as −1 and subjects in the control group as 1) regarding NSC’s indirect mediation effect. These corresponded to 0.01 and 0.39, respectively, and included zero, meaning the difference was not statistically significant. Consequently, the mediation effects of NSC were verified for the influence of warm scent and not for cool scent or control on prosocial behavioral intentions. The effects of age and gender were confirmed through ANCOVA, and the results were not significant (ps > 0.1).

#### 3.2.3. Discussion 

The experiment demonstrated that the experience of warm scent (vs. cool vs. control) positively affects social connectedness, which induces a higher participation in prosocial behavior. Specifically, concepts associated with warmth activated by the experience of warm scents influenced the judgment of the subjects, which can be traced to the warm group’s significantly higher NSC than that of the co and control groups. Thus, warm scents lead to prosocial behavior because of the activation of concepts related to the metaphors of warmth.

In Experiment 2, we examined whether these mechanisms are possible in the presence of actual scents and in circumstances where such a scent is simulated through visual stimulation.

### 3.3. Experiment 2

The purpose of this experiment was to investigate whether a simulation of a scent created through a computer, in a situation where there is no actual scent, affects prosocial behavior. This experiment aimed to confirm first whether a simulation of olfactory stimulation via a visual stimulation (i.e., multimodal approach) evokes concepts associated with warmth. Subsequently, NSC was measured using a method that was different from that in Experiment 1, wherein it was measured as a mediator. The mediation effect was verified using the Inclusion of Other in the Self (IOS) scale, which is a measure of social connectedness proposed by Ng and Lai [49]. Prosocial behavior, which was used as the dependent variable, was defined as the intention to purchase eco-friendly products. In the previous experiment, we examined the effect of warm scent using comparisons among warm, cool, and control conditions. In everyday life, the word ‘cool’ is often used to describe a person’s personality or appearance in a positive way (e.g., ‘you are so cool’). Because we manipulated the difference in temperature participants experienced only through simulation through language, we describe a situation that contrasts, with ‘warm’ as ‘cold’ rather than ‘cool’, in order to control for possible side effects relating to the meaning of ‘cool’.

#### 3.3.1. Methods

##### Participants and Design

A total of 995 participants (male: 612; female: 383) were recruited from Amazon Mechanical Turk (Mturk) (ages 18 to 70 years; M = 32.94, SD = 10.33). The experiment adopted a between-subjects design (scent: warm vs. cold vs. control). The dependent variable was the intention to participate in prosocial behavior, and the mediator was NSC.

##### Procedure and Materials

**Figure A2:**
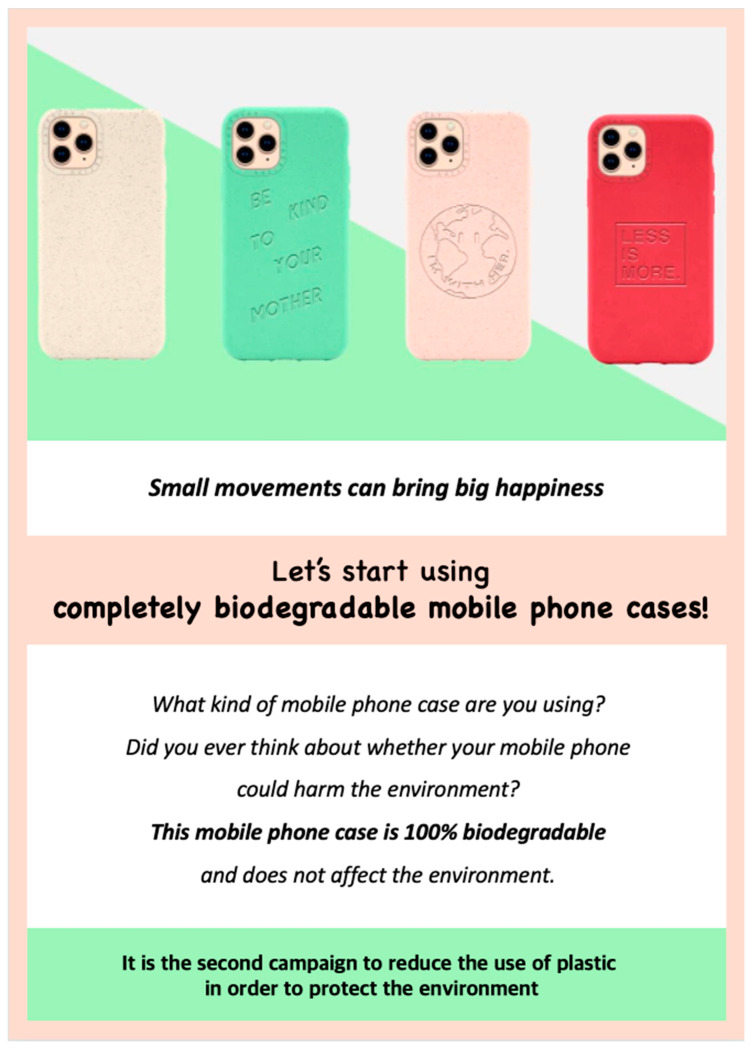
Intentions to purchase an eco-friendly product (Experiment 2).

**Figure A3:**
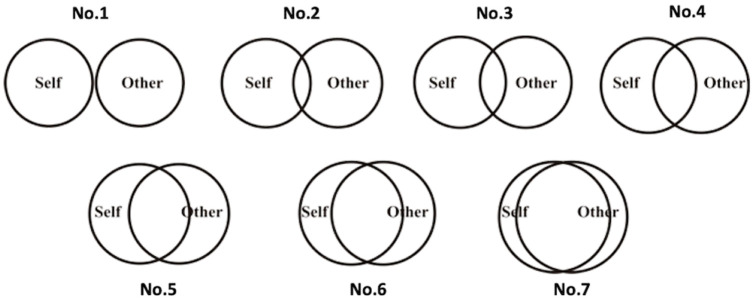
IOS: Inclusion of Other in the Self scale.

First, all subjects participated in an unrelated study that was irrelevant to the experiment. Later, the warm group was instructed to read the types of warm scents, along with their simple descriptions to facilitate the imagination of a warm scent (e.g., “Cozy vanilla scents make your mood comfortable and soft”). The cold group was also instructed to read the types of cold scents and descriptions (e.g., “The chilly peppermint scent is sharp and makes you feel like someone gave you a cold shoulder”). Then, using adjectives related to warmth and cold (vs. neutral), a manipulation check was conducted by measuring how the participants felt about the descriptions of the scents that they had read (warmth-related adjectives: −3 = finicky vs. 3 = mild, −3 = touchy vs. 3 = peaceful, −3 = unfamiliar vs. 3 = familiar; cold-related adjectives: −3 = unfriendly vs. 3 = friendly, −3 = indifferent vs. 3 = concerned, −3 = nippy vs. 3 = boiling; neutral-related adjective: −3 = boring vs. 3 = funny, −3 = cowardly vs. 3 = courageous, −3 = inauthentic vs. 3 = authentic). Next, participants from all three groups responded on a seven-point scale to two questions regarding intentions to purchase an eco-friendly product (see Figure A2; −3 = I do not want to purchase the product at all vs. 3 = I want to purchase the product very much). Furthermore, to measure NSC based on the IOS scale (Figure A3) [50], the subjects were instructed to select an image that was close to their interpersonal relationships. In the process, 18 respondents who omitted one or two response questions from the questionnaire were removed. Finally, the subjects were asked to provide their gender and age. After all experimental procedures had been concluded, the subjects were provided with rewards, along with a message of appreciation.

#### 3.3.2. Results

##### Manipulation Check

In the independent sample t-test, which was conducted with 361 and 249 subjects in the warm and cold groups, respectively, the difference between the two types of adjectives was found to be significant (warm group: M_warmth-related adjectives_ = 5.13 vs. M_neutral-related adjectives_ = 4.94; *t*(360) = 2.02, *p* < 0.05; cold group: M_cold-related adjectives_ = 3.97 vs. M_neutral-related adjectives_ = 4.54; *t*(360) = −5.98, *p* < 0.001, CI = 0.00, 0.36), indicating a successful manipulation of warmth.

##### Experimental Results

To determine the results, a one-way ANOVA was conducted with scent (warm vs. cold vs. control) as an independent variable and prosocial behavioral intention as a dependent variable. The results demonstrated greater intention to participate in prosocial behavior in the group exposed to the warm scent (M_warm_ = 5.75, SD = 1.13 vs. M_cold_ = 5.44, SD = 1.44 vs. M_control_ = 5.48, SD = 1.29; *F*(2, 992) = 5.95, *p* < 0.01, CI = 5.49, 5.65′ see Figure 2). Specifically, a planned contrast that was conducted with the warm group’s prosocial behavior intention was higher than that of the cold group ([t(992) = 2.95, *p* < 0.01, Cohen’s *d* = 0.24 (< 0.02, small)]) and the control group ([t(992) = 2.93, *p* < 0.01, Cohen’s d = 0.22 (<0.02, small)]). The difference between the cold group and the control group was not significant (*p* > 0.1).

An analysis using PROCESS model No. 4 by Hayes [51] was conducted to examine whether the NSC measured by the IOS scale mediated the effects of warm scents on prosocial behavior. Warm scent (with subjects in the warm group coded as 1 and subjects in the cold group as −1) was the independent variable; intentions of prosocial behavior (i.e., intentions to purchase eco-friendly products) were the dependent variables; and “inclusion of the other in the self” was the mediator. The number of samples re-extracted for the bootstrap was 10,000, and the LLCI and ULCI of the mediation effect coefficient were obtained from a 95% CI. First, the results of the intention of prosocial behavior did not include zero between the LLCI and ULCI of the indirect effect, which were 0.02 and 0.06, respectively, thus verifying statistical significance (For direct effect, see Table 3). As for the results of the cold and control groups (with subjects in the cold group coded as −1 and subjects in the control group as 1), zero was likewise not included between the LLCI and ULCI, which were 0.04 and 0.13, respectively, similarly demonstrating statistically significant results. Therefore, the mediation effect of “inclusion of the other in the self” in terms of the effects of warm scents on prosocial behavior, was validated (see Table 4). However, for cold vs. control, this process was not significant. The effects of age and gender were confirmed through ANCOVA, and the results were not significant (ps > 0.1).

#### 3.3.3. Discussion

The results of Experiment 2 showed that the positive effects of warm scents on prosocial behavior are possible even by the mere simulation of scents in an environment where such scents are absent. This result is significant, as it validates that the concepts related to metaphors can be roused not just by olfactory stimuli, but also that a mere simulated scent can activate metaphor-related concepts based on a visual stimulus (i.e., a multimodal approach).

## 4. General Discussion

### 4.1. Theoretical Contributions

In this study, a simple and intuitive method that can promote the need for social connectedness and ensure sustainable social wellbeing was explored, and a solution was presented. In particular, the mechanism tested in our study shows that the activation of the metaphor for warmth raises the need for social connectedness and leads to prosocial behavior.

The current study looked at prosocial behavior through prosocial spending and participation in prosocial campaigns, and such behaviors that have been shown to foster a sense of belonging for both the offeror and the receiver of social support [52]. According to previous literature, social satisfaction was found in both prosocial spending and prosocial activities, including campaign activities, such as volunteering and product donations [53,54].

Furthermore, this study is significant in that it expanded the use of multimodality, which had been mainly used in the context of image or voice recognition, to olfactory stimulation research. In particular, the effect of the multimodal approach using visual stimulus-based olfactory simulation was observed to be significant, and it is expected that this effect will be exploited by ESG (Environmental, Social, and Governance) initiatives in the current pandemic situation. This effect was verified through experiments showing similar reactions to both simulated and actual scents. In other words, it was found that the NSC occurred equally in the warm surrounding environment and the simulated warm environment, leading to prosocial behavior.

Through a series of experiments, our findings indicate that an olfactory stimulus can effectively convince consumers to display behaviors related to embodied metaphors, when perceived as actual and imaginary scents. All in all, the experience of a warm scent with a metaphor can positively influence an individual’s prosocial thinking, which can, for example, induce one’s voluntary participation in sustainable prosocial marketing, thereby contributing to the prosocial development of the community.

### 4.2. Limitations and Future Direction of Research

Four limitations warrant discussion, which leads to implications for future studies.

First, in this study, it was examined that warm behavior can be induced through a warm scent. As it has been found that a similar effect appears in sweetness, we propose that future research on metaphors related to sweetness should also be carried out. Sweetness has been generally studied using the sense of taste and not that of smell and has been found to give prosocial behaviors positive effects. It has also been found to assess other more lenient scents that are similar to warm scents. Second, we argue that the range of temperature may be different. Although the metaphor of warmth activates concepts like kindness and caring, similar thermal expressions such as hot, heat, and burning may be interpreted differently depending on cultural and linguistic differences (e.g., hot can express the attractiveness the one feels) [55]. Therefore, in this study, we authors propose to explore this field in a future study.

The main purpose of this study is to investigate the effect of warmth, and yet “peppermint scent,” which is classified as a cool scent, was used. Additionally, the effect of its scent was not separately investigated. This is one of the potential limitations of the study. However, since various studies related to the metaphor of ‘cool’ are being conducted, it would be reasonable to look at these aspects together in future studies. Another limitation is that the age of the subjects in the actual-scent experiment was relatively low. Olfactory function is known to decrease with age [56]. Thus, it is considered that the results of the experiment may show different patterns due to changes in the olfactory threshold, according to age. We hope that further research will provide greater insights into these issues.

## Figures and Tables

**Table 1:** Direct Effect between the Experience of Warmth and Intentions toward Prosocial Behavior.

	Intention to Participate in a Bag-Donation Campaign				
	*β*	SE	*t*	Boot ULCI	Boot ULCI
Warmth of scent (vs. Cool)	0.28	0.15	1.55	−0.06	0.54
Need for social connectedness	0.64	0.16	3.79 ***	0.30	0.98

Note: * *np* < 0.05, ** *p* < 0.01, *** *p* < 0.001.

**Table 2:** Mediation Effect of the Need for Social Connection in the Relationship between the Experience of Warmth and Intentions toward Prosocial Behavior.

Mediating Factor	Indirect Effect	Bootstrap Standard Error	95% Confidence Interval	
			Boot ULCI	Boot ULCI
Need for social connection	0.15	0.09	0.03	0.37

**Table 3:** Direct Effect between the Experience of Warmth and Intentions toward Prosocial Behavior.

	Intention to Purchase Eco-Friendly Products				
	*β*	SE	*t*	Boot ULCI	Boot ULCI
**Warmth of scent (vs. Cold)**	0.10	0.05	2.04 *	0.00	0.21
**Need for social connectedness**	0.11	0.02	4.22 ***	0.06	0.17

Note: * *p* < 0.05, ** *p* < 0.01, *** *p* < 0.001.

**Table 4:** Mediation Effect of Inclusion of the Other in the Self in the Relationship between the Experience of Warmth and Intentions toward Prosocial Behavior.

Mediating Factors	Indirect Effects	Bootstrap Standard Error	95% Confidence Interval	
			Boot ULCI	Boot ULCI
**Inclusion of the other in the self**	0.04	0.01	0.02	0.06

## Data Availability

Data collected and analyzed during the study are available upon reasonable request.
